# Association of Variation in Behavioral Symptoms With Initial Cognitive Phenotype in Adults With Dementia Confirmed by Neuropathology

**DOI:** 10.1001/jamanetworkopen.2022.0729

**Published:** 2022-03-03

**Authors:** Jagan A. Pillai, James Bena, Kasia Rothenberg, Bryce Boron, James B. Leverenz

**Affiliations:** 1 Lou Ruvo Center for Brain Health, Cleveland Clinic, Cleveland, Ohio; 2 Neurological Institute, Cleveland Clinic, Cleveland, Ohio; 3 Department of Neurology, Cleveland Clinic, Cleveland, Ohio; 4 Quantitative Health Sciences, Cleveland Clinic, Cleveland, Ohio; 5 Department of Psychiatry, Cleveland Clinic, Cleveland, Ohio

## Abstract

**Importance:**

Behavioral and psychological symptoms of dementia (BPSDs) in association with amnestic and nonamnestic cognitive phenotypes have not been evaluated across diagnoses of Alzheimer disease pathology (ADP), Lewy body–related pathology (LRP), and mixed pathology (ADP-LRP).

**Objectives:**

To determine the clinical phenotypes at the initial visit that are associated with the nature and severity of BPSDs in patients with ADP, LRP, and ADP-LRP.

**Design, Setting, and Participants:**

This retrospective longitudinal cohort study included 2422 participants with neuropathologically confirmed ADP, LRP, or mixed ADP-LRP in the National Alzheimer Coordinating Center database from June 20, 2005, to September 4, 2019. Participants had a mean (SD) interval of 5.5 (2.8) years from initial visit to autopsy.

**Main Outcomes and Measures:**

Clinician-determined diagnosis of change across 10 BPSDs (agitation, apathy, depression, delusions, disinhibition, auditory hallucinations, visual hallucinations, irritability, personality change, and rapid eye movement [REM] sleep behavior) and the highest severity score for behavioral change on the Neuropsychiatric Inventory Questionnaire (NPI-Q).

**Results:**

A total of 2422 participants (1187 with ADP, 904 with ADP-LRP, and 331 with LRP) were included in the analysis (1446 men [59.7%]; mean [SD] age, 74.4 [10.1] years). Compared with initial amnestic symptoms, executive symptoms were associated with a higher risk for 7 of the 10 BPSDs (hazard ratio [HR] range, 1.28-2.45), and visuospatial symptoms were associated with a higher risk for 2 of the 10 BPSDs (HR range, 1.91-2.51), but neither were associated with a low risk for any BPSD. Language symptoms were associated with a low risk of onset for 3 of 10 BPSDs (HR range, 0.43-0.79) and a high risk for 1 BPSD (personality change) (HR, 1.42 [95% CI, 1.10-1.83]). Participants with LRP had a lower risk for agitation (HR, 0.74 [95% CI, 0.60-0.92]), disinhibition (HR, 0.78 [95% CI, 0.62-0.99]), and irritability (HR, 0.81 [95% CI, 0.68-0.96]) and a higher risk for apathy (HR, 1.19 [95% CI, 1.02-1.38]), depression (HR, 1.32 [95% CI, 1.12-1.55]), auditory (HR, 2.00 [95% CI, 1.37-2.93]) and visual (HR, 2.78 [95% CI, 2.21-3.49]) hallucinations, and REM sleep behavior changes (HR, 4.77 [95% CI, 3.61-6.31]) compared with the ADP group. The ADP-LRP group had a higher risk for delusions (HR, 1.27 [95% CI, 1.08-1.48]), auditory (HR, 1.62 [95% CI, 1.21-2.15]) and visual (HR, 1.57 [95% CI, 1.30-1.89]) hallucinations, and REM sleep behavior changes (HR, 2.10 [95% CI, 1.63-2.70]) than the ADP group and a lower risk for visual hallucinations (HR, 0.56 [95% CI, 0.45-0.71]) and REM sleep behavior changes (HR, 0.44 [95% CI, 0.34-0.57) than the LRP group. Overall, women showed a lower risk of agitation (HR, 0.86 [95% CI, 0.75-0.98]), apathy (HR, 0.79 [95% CI, 0.71-0.87]), visual hallucinations (HR, 0.76 [95% CI, 0.64-0.90]), irritability (HR, 0.77 [95% CI, 0.69-0.86]), and REM sleep behavior change (HR, 0.45 [95% CI, 0.35-0.58]) and a higher risk of depression (HR, 1.26 [95% CI, 1.13-1.41]). Older age was associated with a lower risk of most BPSDs (HR range, 0.98-0.99) except delusions (HR, 1.00 [95% CI, 1.00-1.01]) and auditory hallucinations (HR, 0.99 [95% CI, 0.97-1.00]) and a low NPI-Q composite score (β = −0.07 [95% CI, −0.08 to −0.05]; *P* < .001).

**Conclusions and Relevance:**

These findings suggest that the risks of BPSDs differ with respect to the initial cognitive phenotype, underlying neuropathology, age, and sex. Awareness of these associations could be helpful in dementia management.

## Key Points

**Question:**

Are the initial cognitive phenotypes associated with risk of behavioral and psychological symptoms of dementia (BPSDs) in patients with Alzheimer disease pathology (ADP), Lewy body–related pathology (LRP), and mixed ADP-LRP?

**Findings:**

In this cohort study of 2422 participants, executive and visuospatial symptoms were associated with a higher risk for specific BPSDs compared with amnestic symptoms, whereas language phenotype was associated with a lower risk for some BPSDs. The ADP-LRP group had a higher risk for delusions, auditory and visual hallucinations, and rapid eye movement sleep behavior changes than the ADP group but a lower risk for visual hallucinations and rapid eye movement sleep behavior changes than the LRP group.

**Meaning:**

These findings suggest that initial cognitive phenotype and underlying neuropathology are associated with subsequent risk for specific BPSDs.

## Teaser

This cohort study assesses whether different initial cognitive phenotypes have distinct associated behavioral and psychological symptoms of dementia and whether the risk of onset for specific symptoms differs by neuropathology in the clinical course among patients with dementia.

## Introduction

Behavioral and psychological symptoms of dementia (BPSDs) are an important feature in the clinical course of all types of dementia.^1^ Behavioral and psychological symptoms of dementia are associated with dementia severity and progression,^2^ functional limitations,^3^ earlier institutionalization,^4,5^ and greater mortality,^6^ as well as greater caregiver burden.^7,8^ Given the current limitations of effective therapies for dementia and related BPSDs,^9^ a better understanding of the clinical and pathological factors associated with the onset of BPSDs in the dementia syndromes may be useful for clinicians in caring for patients with dementia and counseling caregivers. Behavioral and psychological symptoms of dementia have been discussed widely in Alzheimer disease (AD) owing to its high prevalence,^10,11^ but BPSDs are also common in dementia with Lewy bodies and other dementias.^12,13,14,15,16,17^ The specific factors that predispose an individual to the assortment of BPSDs in each of these dementia syndromes is an area of significant interest.

The occurrence of BPSDs has been investigated in the complex interplay of social, psychological, and biological factors.^18,19,20^ The classical paradigm in molecular neuropsychiatry is that the behavioral phenotype is linked through selective regional vulnerability of neuronal populations for each specific neuropathology, with the topography of the neuronal dysfunction, in turn, determining the clinical phenotype.^21^ Identification of regional brain changes associated with neurodegeneration and relation of these changes to each specific BPSD^22,23,24^ and its association with the occurrence of future BPSDs is of interest.^25^ However, heterogeneity exists among initial predominant clinical symptoms in primary AD pathology (ADP)^26^ and Lewy body–related pathology (LRP)^27,28^ in addition to mixed ADP-LRP^29^ in dementia. Biomarker differences among patients with AD and dementia with Lewy bodies with predominant amnestic and nonamnestic symptoms (predominant initial symptoms in language, executive, or visuospatial domains),^28,30,31^ along with regional patterns of neurofibrillary tangle accumulation^32^ and Lewy body pathology,^27^ suggest that underlying pathological characteristics could influence early presentations of the initial clinical symptoms. Therefore, the topography of neuronal dysfunction noted in cognitive phenotypes and the underlying specific BPSDs may overlap. However, how the initial cognitive presentation is associated with the risk of subsequent BPSDs among different neuropathological subsets is unknown. Moreover, even as multiple neurodegenerative pathological features are thought to lower the threshold for clinical dementia,^33^ the impact of mixed pathology on BPSDs is unclear.

This study focuses on ADP and LRP, which have well-known differences in regional distribution of neuropathology,^34,35,36^ as well as mixed ADP-LRP. Our main hypotheses were that (1) different initial cognitive phenotypes have distinct associated BPSDs and (2) the risk of onset for specific BPSDs differs by neuropathology in the clinical course of dementia.

## Methods

### Participants and Study Design

This retrospective longitudinal cohort study used the National Alzheimer Coordinating Center (NACC) database and included participant information collected from 37 past and present AD centers funded by the National Institute on Aging. Data from the Uniform Data Set maintained by the NACC between June 20, 2005, and September 4, 2019, were analyzed. All contributing AD centers are required to obtain written informed consent from their participants and maintain their own separate evaluation and approval from their institutional review board before submitting data to the NACC. Details on data collection and data curating are well documented^37^ (eMethods in the Supplement). This study followed the Strengthening the Reporting of Observational Studies in Epidemiology (STROBE) reporting guideline.

### Participant Assessment and Inclusion Criteria

All participants had ADP (tau neurofibrillary tangle pathology Braak stages III-VI and moderate or frequent neuritic plaques) or LRP (brainstem predominant, limbic or amygdala predominant, neocortical, or Lewy bodies present but region unspecified). The ADP group was defined by underlying ADP without coexisting LRP, and the LRP group had underlying LRP without concomitant ADP. The ADP-LRP group was defined when pathological features of both diseases were documented together. Because mixed vascular pathology is expected in many older individuals and the degree of vascular pathology could change from the initial visit to autopsy, all participants were characterized for the likelihood of coexisting vascular pathology by the Hachinski Ischemic Scale when available at the time of initial evaluation for each group^38^ (eMethods and eTable 1 in the Supplement). Measures from the NACC neuropathology data across centers show good agreement.^39^

The Clinical Dementia Rating (CDR) instrument was completed by all participants who had a CDR-Global (CDR-G) score of at least 1 at the initial clinical visit. The CDR-G ratings are calculated using a complex algorithm and range from 0 (no dementia) to 3 (severe dementia).^40^ A CDR-G score of 1 corresponds to the threshold of early dementia. We limited analysis to those with CDR-G of at least 1 at the initial visit to increase the reliability of data regarding initial cognitive symptoms closest to their onset, when the dementia symptoms are still early in their trajectory (eMethods in the Supplement). The eFigure in the Supplement provides the participant selection algorithm.

### Initial Cognitive Symptoms and Neuropsychological Tests

The predominant symptom that was first recognized by clinicians as a decline in participant cognition was used in this analysis. This is denoted in the NACC data set as the variable NACCCOGF. The International Working Group–2 research diagnostic criteria define specific clinical phenotypes of frontal, logopenic, and posterior variants of AD, but these cannot be defined based on the first cognitive domain of decline from a clinical impression alone.^26^ Participants were characterized as having primary amnestic (if memory loss was the initial symptom), executive (if executive or attention/concentration dysfunction was the initial symptom), language (if language dysfunction was the initial symptom), or visuospatial (if visuospatial dysfunction was the initial symptom) features. Participants’ neuropsychology data were not used in determining initial symptoms by the clinicians involved. Cognitive profiles noted on neuropsychology evaluations at the initial clinical visit in the NACC cohort have been reported to be congruent with the initial cognitive symptoms as noted by clinicians.^41^ Details on NACCCOGF and neuropsychological measures^42^ are presented in the eMethods in the Supplement.

### Behavioral Symptoms and Neuropsychiatric Inventory Questionnaire

Two BPSD measures were used: a clinician-determined Uniform Data Set score sheet in evaluating the onset of each specific BPSD and a caregiver-determined Neuropsychiatric Inventory Questionnaire (NPI-Q) in determining the burden of all BPSDs taken together for an individual. These measures capture many, but not all, of the same BPSD symptoms (eMethods in the Supplement). Behavioral symptoms for each participant were collated from the clinician-determined NACC-derived variables that indicate whether the “subject currently manifests meaningful change in behavior” after a clinician interview at each visit. The abnormal behaviors noted include agitation, apathy, anxiety, depressed mood, psychosis in the form of visual hallucinations, auditory hallucinations, and abnormal false or delusional beliefs, disinhibition, irritability, personality change, and rapid eye movement (REM) sleep behavior disorder.

The NPI-Q is a briefer questionnaire derived from the NPI^43^ that includes ratings of behavioral domain severity only (not frequency) and was developed for use in general clinical practice or epidemiologic research settings.^44^ The NPI-Q is a caregiver-reported questionnaire that can be completed in 5 minutes or less.^44^ The highest NPI-Q total score for each participant was collated and used as a measure to capture the maximum severity of behavioral symptoms during longitudinal follow-up.

### Statistical Analysis

Data were analyzed from June 20, 2005, to September 4, 2019. Normality was assessed for continuous variables by Q-Q plots. A 2-tailed *t* test was applied to compare normally distributed continuous variables. We performed the χ^2^ test for categorical variables. Given the differential follow-up across participants, time to the first BPSD event was analyzed for each BPSD separately using Cox proportional hazards regression models. Multiple imputation using fully conditional specification was performed to account for missing data in covariates used in the multivariable models (*APOE ε4* status [missing on 235 (9.7%)] and years of education [missing on 17 (0.7%)]), and 10 imputation data sets were created. Models were run separately for each imputation data set and then pooled using Rubin’s rules^45^ to provide overall results. Adequacy of the proportional hazards assumption was evaluated using Schoenfeld residuals. Here, models included timing and occurrence of behavioral symptoms during disease course as outcomes, and *APOE ε4* carrier status (yes or no), initial cognitive symptoms, age, sex, years of education, and the neuropathological group as variables associated with outcomes. In the models, amnestic initial symptoms were used as a reference level and compared with other initial symptoms. Contrasts for all pairwise comparisons of neuropathological groups were included so that all pairwise comparisons are provided. Interaction terms between initial cognitive symptoms and neuropathological groups were first assessed but were not found to be significant after correction for false discovery rate (FDR) in models. Therefore, the results from models without interactions are presented. Sensitivity analyses comparing initial symptoms within pathological groups were completed to assess the robustness of the results.

Correction for FDR at .05 was applied to the results of each BPSD model and is presented along with the uncorrected 95% CIs. Healthy control participants with longitudinal follow-up and behavioral changes with neuropathological features were limited and are not included in the models.

Next, to identify factors that are associated with the severity of NPI-Q scores during the subsequent clinical course, multivariable linear mixed-effect regression models (identity link, normal error distribution) for all participants taken together and within each of the neuropathological groups were assessed in separate analyses. The composite NPI-Q score for each participant was the dependent variable, with age, sex, educational level, *APOE ε4* genotype, initial cognitive symptoms, and neuropathological group as the independent variables. Interactions between time and neuropathological group were not significant, so only a main-effect model is provided. We calculated 95% CIs of the β estimates and performed collinearity diagnostics. As above, the amnestic initial symptom was taken as the reference group, and in the full model, contrasts were used to compare all pairs of neuropathological groups.

All tests were 2 tailed and were performed at an overall significance level of *P* < .05. We used R, version 3.5.1 (R Foundation for Statistical Computing) and SPSS Statistics for Windows, version 22.0 (IBM Corp), for analyses.

## Results

A total of 2422 participants were included in the analysis (976 women [40.3%] and 1446 men [59.7%]; mean [SD] age, 74.4 [10.1] years). A total of 4 participants (0.2%) were American Indian or Native Hawaiian; 18 (0.8%), Asian; 93 (3.9%), Black; 2296 (95.1%), White; and 3 (0.1%), other race. Given the low numbers of non-White races in this cohort with neuropathology-confirmed dementia, the impact of race on the hypotheses of interest could not be meaningfully assessed owing to lack of power. Of these 2422 participants, 1187 had ADP, 904 had ADP-LRP, and 331 had LRP. The mean (SD) time from initial visit to autopsy was 5.5 (2.8) years. Table 1 provides demographic characteristics of the participants in the NACC database and their clinical features of interest. Significant differences across groups were observed for all characteristics except educational level (*F* = 0.5; *P* = .60), the development of all behavior changes except apathy (χ^2^ = 5.6; *P* = .06) and personality change (χ^2^ = 0.2; *P* = .91), and maximum NPI-Q score (*F* = 2.1; *P* = .12). Given the smaller number of participants with anxiety in the LRP group, model analysis results for anxiety are of limited utility and are provided in the eMethods and eTable 2 in the Supplement.

**Table 1.** Participant Demographics for Each Neuropathology Group and Nature of Initial Cognitive Symptoms and Behavioral Changes

Characteristic	Neuropathology group						Statistical value	*P* value
	ADP		LRP		ADP-LRP			
	No. of participants	Values	No. of participants	Values	No. of participants	Values		
Age at first visit, mean (SD), y	1187	75.2 (10.3)	331	74.9 (9.6)	904	73.1 (10.0)	*F* = 12.5	<.001^a^
No. of visits, median (IQR)	1187	4.0 (2.0-6.0)	331	3.0 (2.0-5.0)	904	4.0 (2.0-6.0)	*F* = 6.2	.002^a^
Time to follow-up visit, mean (SD), y	1187	3.8 (2.7)	331	3.1 (2.5)	904	3.7 (2.7)	*F* = 7.6	<.001^a^
Follow-up to death, mean (SD), y	1187	5.6 (2.7)	331	4.7 (2.8)	904	5.8 (2.9)	*F* = 19.6	<.001^a^
Age at first visit, mean (SD), y	1187	80.8 (10.3)	331	79.6 (9.7)	904	78.8 (10.1)	*F* = 9.7	<.001^a^
Educational level, mean (SD), y	1177	15.5 (3.0)	330	15.6 (3.2)	898	15.6 (3.1)	*F* = 0.5	.60^a^
Sex, No. (%)								
Women	1187	543 (45.7)	331	90 (27.2)	904	343 (37.9)	χ^2^ = 41.4	<.001^b^
Men		644 (54.3)		241 (72.8)		561 (62.1)		
*APOE* ε4, No. (%)	1072	626 (58.4)	289	100 (34.6)	826	517 (62.6)	χ^2^ = 70.4	<.001^b^
Hachinski Ischemic Scale score, mean (SD)^c^	1162	0.90 (1.3)	323	1.4 (1.8)	870	0.82 (1.3)	*F* = 19.0	<.001^a^
Initial symptoms, No. (%)								
Amnestic	1187	934 (78.7)	331	212 (64.0)	904	721 (79.8)	χ^2^ = 50.1	<.001^b^
Executive		91 (7.7)		59 (17.8)		70 (7.7)		
Language		130 (11.0)		36 (10.9)		80 (8.8)		
Visuospatial		32 (2.7)		24 (7.3)		33 (3.7)		
BPSD changes, mean (SD)								
Agitation	1187	531 (44.7)	331	105 (31.7)	904	389 (43.0)	χ^2^ = 18.7	<.001^b^
Anxiety	1187	119 (10.0)	331	22 (6.6)	904	106 (11.7)	χ^2^ = 7.4	.03^b^
Apathy	1187	846 (71.3)	331	242 (73.1)	904	686 (75.9)	χ^2^ = 5.6	.06^b^
Depression	1187	645 (54.3)	331	203 (61.3)	904	550 (60.8)	χ^2^ = 10.9	.004^b^
Delusions	1187	328 (27.6)	331	80 (24.2)	904	306 (33.8)	χ^2^ = 14.7	<.001^b^
Disinhibition	1187	439 (37.0)	331	90 (27.2)	904	315 (34.8)	χ^2^ = 11.3	.004^b^
Auditory hallucinations	1187	86 (7.2)	331	43 (13.0)	904	106 (11.7)	χ^2^ = 16.6	<.001^b^
Visual hallucinations	1187	208 (17.5)	331	130 (39.3)	904	248 (27.4)	χ^2^ = 72.8	<.001^b^
Irritability	1187	701 (59.1)	331	162 (48.9)	904	531 (58.7)	χ^2^ = 11.5	.003^b^
Personality change	1187	311 (26.2)	331	90 (27.2)	904	243 (26.9)	χ^2^ = 0.2	.91^b^
REM sleep change	1187	98 (8.3)	331	116 (35.0)	904	157 (17.4)	χ^2^ = 134.1	<.001^b^
Maximum NPI-Q score, mean (SD)^d^	1187	8.7 (5.8)	331	8.9 (6.2)	904	9.2 (5.7)	*F* = 2.1	.12^a^

Abbreviations: ADP, Alzheimer disease pathology; *APOE*, apolipoprotein E; BPSD, behavioral and psychological symptoms of dementia; LRP, Lewy body–related pathology; NPI-Q, Neuropsychiatric Inventory Questionnaire; REM, rapid eye movement.

^a^ Calculated using analysis of variance.

^b^ Calculated using Pearson χ^2^ test.

^c^ Scores range from 0 to 18, with scores less than 4 suggesting primary dementia syndrome without significant vascular contribution.

^d^ Scores range from 0 to 36, with higher scores indicating greater neuropsychiatric symptom severity.

### Model for Risk of BPSD Onset for All Participants

**Figure 1.**
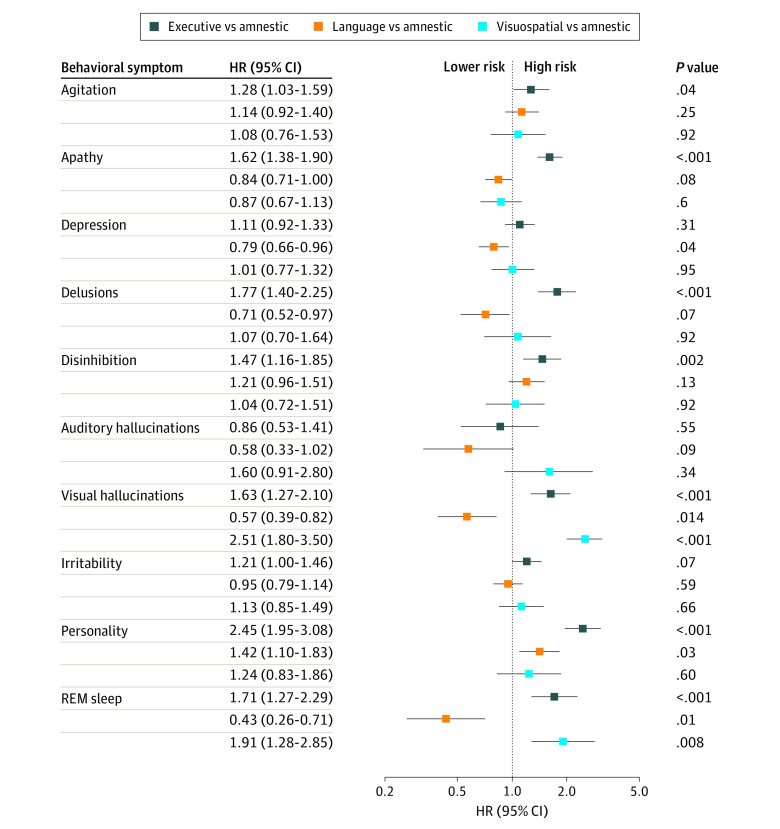
Adjusted Hazards for Each Behavioral and Psychological Symptom of Dementia by Initial Cognitive Symptoms for All Participants Behavioral symptoms include executive, language, and visuospatial dysfunction, with amnestic dysfunction as the reference. HR indicates hazard ratio. *P* values are adjusted for false discovery rate.

Relative to initial amnestic cognitive symptoms, initial language cognitive symptoms were associated with lower risk of depression (HR, 0.79 [95% CI, 0.66-0.96]), visual hallucinations (HR, 0.57 [95% CI, 0.39-0.82]), and REM sleep behavior change (HR, 0.43 [95% CI, 0.26-0.71]) and higher risk of personality changes (HR, 1.42 [95% CI, 1.10-1.83]). There was evidence of lower risk of delusions and apathy, but these did not meet the FDR threshold. Initial executive cognitive symptoms were associated with a higher risk of all BPSDs (eg, HR for delusions, 1.77 [95% CI, 1.40-2.25]; HR for personality changes, 2.45 [95% CI, 1.95-3.08]), except depression (HR, 1.11 [95% CI, 0.92-1.33]), auditory hallucinations (HR, 0.86 [95% CI, 0.53-1.41]), and irritability (HR, 1.21 [95% CI, 1.00-1.46]) relative to amnestic symptoms. Participants with initial visuospatial symptoms were at higher risk of visual hallucinations (HR, 2.51 [95% CI, 1.80-3.50]) and REM sleep behavior changes (HR, 1.91 [95% CI, 1.28-2.85]) than those with initial amnestic symptoms (Figure 1 and Table 2).

**Table 2.** Cox Proportional Hazards Regression Model With All Factors Included as Fixed Effects

Characteristic	BPSD, adjusted HR (95% CI)									
	Agitation	Apathy	Depression	Delusions	Disinhibition	Auditory hallucinations	Visual hallucinations	Irritability	Personality change	REM sleep behavior change
Age at first visit	0.99 (0.98-0.99)^a^	0.99 (0.98-0.99)^a^	0.98 (0.97-0.98)^a^	1.00 (1.00-1.01)	0.98 (0.98-0.99)^a^	0.99 (0.97-1.00)	0.99 (0.98-1.00)^a^	0.99 (0.98-1.00)^a^	0.98 (0.98-0.99)^a^	0.98 (0.97-0.99)^a^
Female sex	0.86 (0.75-0.98)^a^	0.79 (0.71-0.87)^a^	1.26 (1.13-1.41)^b^	0.94 (0.81-1.10)	0.97 (0.84-1.11)	0.90 (0.68-1.17)	0.76 (0.64-0.90)^a^	0.77 (0.69-0.86)^a^	0.87 (0.74-1.02)	0.45 (0.35-0.58)^a^
Educational level	1.00 (0.98-1.02)	0.98 (0.97-1.00)^c^	1.00 (0.98-1.01)	0.97 (0.95-1.00)^c^	0.99 (0.97-1.01)	0.95 (0.92-1.00)^c^	0.96 (0.93-0.98)^a^	0.97 (0.96-0.99)^a^	0.97 (0.95-1.00)^c^	1.01 (0.98-1.05)
*APOE* ε4	1.01 (0.88-1.15)	1.04 (0.94-1.15)	0.98 (0.87-1.11)	1.14 (0.96-1.35)	1.00 (0.86-1.16)	0.86 (0.64-1.14)	0.96 (0.80-1.14)	1.04 (0.93-1.16)	1.20 (1.01-1.43)^d^	0.76 (0.60-0.94)^c^
Executive vs amnestic dysfunction	1.28 (1.03-1.59)^b^	1.62 (1.38-1.90)^b^	1.11 (0.92-1.33)	1.77 (1.40-2.25)^b^	1.47 (1.16-1.85)^b^	0.86 (0.53-1.41)	1.63 (1.27-2.10)^b^	1.21 (1.00-1.46)	2.45 (1.95-3.08)^b^	1.71 (1.27-2.29)^b^
Language vs amnestic dysfunction	1.14 (0.92-1.40)	0.84 (0.71-1.00)^c^	0.79 (0.66-0.96)^a^	0.71 (0.52-0.97)^c^	1.21 (0.96-1.51)	0.58 (0.33-1.02)	0.57 (0.39-0.82)^a^	0.95 (0.79-1.14)	1.42 (1.10-1.83)^b^	0.43 (0.26-0.71)^a^
Visuospatial vs amnestic dysfunction	1.08 (0.76-1.53)	0.87 (0.67-1.13)	1.01 (0.77-1.32)	1.07 (0.70-1.64)	1.04 (0.72-1.51)	1.60 (0.91-2.80)	2.51 (1.80-3.50)^b^	1.13 (0.85-1.49)	1.24 (0.83-1.86)	1.91 (1.28-2.85)^b^
LRP vs ADP groups	0.74 (0.60-0.92)^a^	1.19 (1.02-1.38)^b^	1.32 (1.12-1.55)^b^	0.96 (0.75-1.23)	0.78 (0.62- 0.99)^a^	2.00 (1.37-2.93)^b^	2.78 (2.21-3.49)^b^	0.81 (0.68-0.96)^a^	1.09 (0.86-1.39)	4.77 (3.61-6.31)^b^
ADP-LRP vs ADP groups	0.93 (0.81-1.06)	1.11 (1.00-1.23)^d^	1.13 (1.01-1.27)^d^	1.27 (1.08-1.48)^b^	0.93 (0.80-1.07)	1.62 (1.21-2.15)^b^	1.57 (1.30-1.89)^b^	0.99 (0.88-1.10)	0.98 (0.83-1.17)	2.10 (1.63-2.70)^b^
ADP-LRP vs LRP groups	1.25 (1.00-1.56)^d^	0.93 (0.80-1.09)	0.86 (0.73-1.02)	1.32 (1.02-1.70)^d^	1.19 (0.93-1.51)	0.81 (0.56-1.17)	0.56 (0.45-0.71)^a^	1.22 (1.02- 1.47)^d^	0.90 (0.70-1.16)	0.44 (0.34-0.57)^a^

Abbreviations: ADP, Alzheimer disease pathology; *APOE*, apolipoprotein E; BPSD, behavioral and psychological symptom of dementia; FDR, false discovery rate; HR, hazard ratio; LRP, Lewy body–related pathology; REM, rapid eye movement.

^a^ Indicates *P* < .05 meeting false discovery rate (FDR) threshold for lower HR.

^b^ Indicates *P* < .05 meeting FDR threshold for higher HR.

^c^ Indicates *P* < .05 not meeting FDR threshold for lower HR.

^d^ Indicates *P* < .05 not meeting FDR threshold for higher HR.

**Figure 2.**
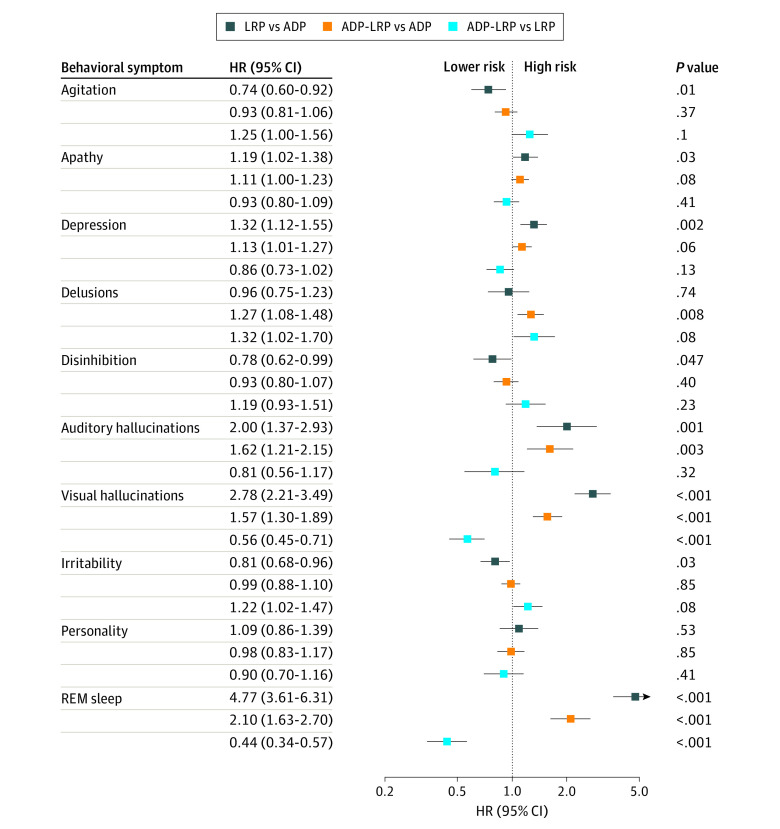
Adjusted Hazards Among Neuropathology Groups for Each Behavioral and Psychological Symptom of Dementia ADP indicates Alzheimer disease pathology; HR, hazard ratio; LRP, Lewy body–related pathology. *P* values are adjusted for false discovery rate.

After accounting for the FDR adjustment, the ADP-LRP group was at higher risk of delusions (HR, 1.27 [95% CI, 1.08-1.48]), auditory hallucinations (HR, 1.62 [95% CI, 1.21-2.15]), visual hallucinations (HR, 1.57 [95% CI, 1.30-1.89]), and REM sleep behavior changes (HR, 2.10 [95% CI, 1.63-2.70]) than the ADP group and lower risk of visual hallucinations (HR, 0.56 [95% CI, 0.45-0.71]) and REM sleep behavior changes (HR, 0.44 [95% CI, 0.34-0.57) than participants with LRP. Those with LRP were at higher risk of apathy (HR, 1.19 [95% CI, 1.02-1.38]), depression (HR, 1.32 [95% CI, 1.12-1.55]), auditory hallucinations (HR, 2.00 [95% CI, 1.37-2.93]), visual hallucinations (HR, 2.78 [95% CI, 2.21-3.49]), and REM sleep behavior change (HR, 4.77 [95% CI, 3.61-6.31]) and lower risk of agitation (HR, 0.74 [95% CI, 0.60-0.92]), disinhibition (HR, 0.78 [95% CI, 0.62-0.99]), and irritability (HR, 0.81 [95% CI, 0.68-0.96]) relative to participants with ADP (Figure 2 and Table 2).

Increased age was associated with a lower risk of all BPSDs except delusions (HR, 1.00 [95% CI, 1.00-1.01]) and auditory hallucinations (HR, 0.99 [95% CI, 0.97-1.00]), whereas women showed a lower risk of agitation (HR, 0.86 [95% CI, 0.75-0.98]), apathy (HR, 0.79 [95% CI, 0.71-0.87]), visual hallucinations (0.76 [95% CI, 0.64-0.90]), irritability (HR, 0.77 [95% CI, 0.69-0.86]), and REM sleep behavior change (HR, 0.45 [95% CI, 0.35-0.58]) and a higher risk of depression (HR, 1.26 [95% CI, 1.13-1.41]). Higher educational level was associated with lower risk of visual hallucinations (HR, 0.96 [95% CI, 0.93-0.98]) and irritability (HR, 0.97 [95% CI, 0.96-0.99]) (Table 2).

### Model for Risk of BPSD Onset Within Each Neuropathology Group

When evaluated within each of the pathology groups, those with executive symptoms consistently had a higher risk for apathy in ADP (HR, 1.62 [95% CI, 1.27-2.06]), ADP-LRP (HR, 1.57 [95% CI, 1.19-2.07]), and LRP (HR, 1.62 [95% CI, 1.16-2.28]) and personality change in ADP (HR, 2.67 [95% CI, 1.92-3.73]), ADP-LRP (HR, 2.40 [95% CI, 1.61-3.56]), and LRP (HR, 1.93 [95% CI, 1.14-3.27]). However, among language symptoms, we found a lower risk of apathy in ADP (HR, 0.75 [95% CI, 0.59-0.95]), with delusions (HR, 0.53 [95% CI, 0.33-0.85]) and visual hallucinations (HR, 0.52 [95% CI, 0.28-0.97]) missing the FDR threshold, and REM sleep behavior changes in LRP (HR, 0.18 [95% CI, 0.06-0.59], with visual hallucinations (HR, 0.40 [95% CI, 0.17-0.95]) missing the FDR threshold. There was further evidence of a higher risk of personality changes with initial language symptoms in ADP-LRP (HR, 2.04 [95% CI, 1.35-3.07]). Visuospatial symptoms had a higher risk among participants with LRP for auditory (HR, 3.43 [95% CI, 1.48-7.97]) and visual (HR, 2.89 [95% CI, 1.68-5.00]) hallucinations and REM sleep behavior change (HR, 2.56 [95% CI, 1.41-4.64]) (Table 3).

**Table 3.** Cox Proportional Hazards Regression Model With All Factors Included as Fixed Effects for Each Neuropathological Group

Variable by neuropathological group	BPSD, adjusted HR (95% CI)									
	Agitation	Apathy	Depression	Delusions	Disinhibition	Auditory hallucinations	Visual hallucinations	Irritability	Personality change	REM sleep change
Age										
ADP	0.99 (0.98-1.00)	0.99 (0.98-0.99)^a^	0.98 (0.97-0.99)^a^	1.00 (0.98-1.01)	0.98 (0.98-0.99)^a^	0.97 (0.95-0.99)^a^	0.99 (0.97-1.00)	0.99 (0.98-1.00)	0.98 (0.97-0.99)^a^	0.97 (0.95-0.99)^b^
ADP-LRP	0.99 (0.98-1.00)	0.99 (0.98-1.00)	0.98 (0.97-0.98)^a^	1.01 (1.00-1.03)	0.99 (0.98-1.00)^b^	1.01 (0.99-1.03)	1.00 (0.99-1.01)	0.99 (0.98-1.00)	0.99 (0.97-1.00)	0.99 (0.98-1.01)
LRP	0.98 (0.96-1.00)	0.97 (0.96-0.99)^a^	0.99 (0.97-1.00)	0.99 (0.96-1.02)	0.96 (0.94-0.98)^a^	0.96 (0.93-1.00)^b^	0.97 (0.95-0.99)^a^	0.98 (0.96-0.99)^b^	0.97 (0.94-0.99)^a^	0.97 (0.95-0.99)^a^
Female sex										
ADP	0.88 (0.74-1.05)	0.79 (0.69-0.91)^a^	1.33 (1.13-1.56)^c^	1.07 (0.86-1.33)	0.90 (0.74-1.09)	1.17 (0.76-1.80)	0.87 (0.66-1.16)	0.75 (0.64-0.87)^a^	1.02 (0.81-1.28)	0.68 (0.45-1.04)
ADP-LRP	0.79 (0.63-0.98)^b^	0.80 (0.68-0.94)^a^	1.29 (1.08-1.53)^c^	0.90 (0.71-1.15)	1.06 (0.83-1.34)	0.83 (0.56-1.25)	0.81 (0.62-1.06)	0.78 (0.65-0.93)^b^	0.79 (0.60-1.04)	0.40 (0.27-0.60)^a^
LRP	1.05 (0.67-1.63)	0.71 (0.52-0.96)^b^	0.97 (0.70-1.34)	0.64 (0.37-1.13)	1.22 (0.76-1.97)	0.47 (0.19-1.11)	0.56 (0.35-0.90)^a^	0.84 (0.58-1.21)	0.62 (0.36-1.07)	0.36 (0.21-0.64)^a^
*APOE* ε4										
ADP	0.98 (0.82-1.17)	1.03 (0.88-1.19)	0.83 (0.69-0.98)^b^	1.09 (0.85-1.39)	0.94 (0.77-1.15)	0.65 (0.42-1.01)	0.86 (0.64-1.15)	0.95 (0.81-1.10)	1.19 (0.94-1.51)	0.74 (0.48-1.13)
ADP-LRP	1.06 (0.84-1.33)	1.06 (0.90-1.25)	1.16 (0.96-1.41)	1.19 (0.91-1.55)	1.09 (0.86-1.39)	0.94 (0.61-1.44)	0.91 (0.69-1.19)	1.11 (0.92-1.34)	1.05 (0.79-1.39)	0.68 (0.49-0.94)
LRP	1.02 (0.66-1.58)	1.02 (0.76-1.37)	1.09 (0.81-1.48)	1.24 (0.75-2.07)	1.03 (0.65-1.63)	0.86 (0.43-1.74)	1.06 (0.71-1.58)	1.27 (0.91-1.78)	1.91 (1.21-3.03)^c^	0.83 (0.55-1.25)
Educational level										
ADP	0.99 (0.96-1.02)	0.98 (0.95-1.00)	0.98 (0.96-1.01)	0.97 (0.94-1.01)	0.99 (0.96-1.03)	0.95 (0.88-1.02)	0.92 (0.88-0.96)^a^	0.97 (0.94-0.99)^a^	0.96 (0.93-1.00)	1.01 (0.95-1.08)
ADP-LRP	1.00 (0.97-1.03)	1.00 (0.97-1.02)	1.02 (0.99-1.05)	0.98 (0.94-1.01)	1.00 (0.96-1.04)	0.93 (0.88-0.99)^b^	0.97 (0.93-1.01)	0.98 (0.95-1.01)	0.98 (0.94-1.02)	1.01 (0.96-1.06)
LRP	0.99 (0.93-1.06)	0.97 (0.94-1.01)	0.98 (0.94-1.02)	0.96 (0.89-1.02)	0.95 (0.89-1.01)	1.03 (0.93-1.13)	0.99 (0.94-1.05)	0.97 (0.92-1.02)	0.98 (0.92-1.04)	1.03 (0.98-1.09)
Executive vs amnestic dysfunction										
ADP	1.25 (0.90-1.72)	1.62 (1.27-2.06)^c^	1.23 (0.93-1.62)	1.46 (1.00-2.13)	1.87 (1.37-2.55)^c^	0.53 (0.19-1.47)	1.39 (0.86-2.26)	1.52 (1.16-1.98)^c^	2.67 (1.92-3.73)^c^	0.76 (0.33-1.76)
ADP-LRP	1.41 (0.99-2.02)	1.57 (1.19-2.07)^c^	1.17 (0.86-1.60)	1.96 (1.34-2.89)^c^	1.37 (0.90-2.07)	0.82 (0.36-1.90)	1.55 (1.01-2.39)^d^	1.16 (0.84-1.60)	2.40 (1.61-3.56)^c^	2.62 (1.70-4.05)^c^
LRP	1.02 (0.58-1.79)	1.62 (1.16-2.28)^c^	0.90 (0.61-1.34)	2.23 (1.31-3.80)^c^	0.77 (0.40-1.48)	1.44 (0.63-3.27)	1.89 (1.23-2.93)^c^	0.74 (0.45-1.20)	1.93 (1.14-3.27)^c^	1.52 (0.95-2.42)
Language vs amnestic dysfunction										
ADP	1.06 (0.81-1.40)	0.75 (0.59-0.95)^a^	0.85 (0.65-1.11)	0.53 (0.33-0.85)^b^	1.05 (0.77-1.44)	0.43 (0.17-1.10)	0.52 (0.28-0.97)^b^	0.85 (0.65-1.10)	0.96 (0.65-1.42)	0.42 (0.17-1.05)
ADP-LRP	1.10 (0.75-1.63)	0.84 (0.63-1.14)	0.72 (0.52-1.01)	0.92 (0.57-1.49)	1.48 (1.00-2.18)	0.70 (0.28-1.74)	0.75 (0.43-1.31)	0.96 (0.70-1.33)	2.04 (1.35-3.07)^c^	0.68 (0.34-1.35)
LRP	1.41 (0.78-2.56)	1.08 (0.69-1.69)	0.88 (0.54-1.44)	0.82 (0.33-2.00)	1.17 (0.62-2.20)	0.63 (0.18-2.21)	0.40 (0.17-0.95)^b^	1.27 (0.78-2.08)	1.83 (0.96-3.50)	0.18 (0.06-0.59)^a^
Visuospatial vs amnestic dysfunction										
ADP	0.89 (0.51-1.57)	0.65 (0.41-1.04)	0.94 (0.59-1.51)	0.87 (0.42-1.78)	1.04 (0.60-1.80)	0.27 (0.04-1.97)	2.12 (1.10-4.07)^d^	0.96 (0.61-1.51)	1.16 (0.60-2.22)	1.00 (0.35-2.84)
ADP-LRP	1.50 (0.88-2.56)	1.09 (0.73-1.63)	0.93 (0.60-1.45)	1.16 (0.59-2.29)	1.20 (0.65-2.23)	1.42 (0.51-3.95)	1.71 (0.92-3.18)	1.39 (0.89-2.17)	1.61 (0.84-3.09)	1.50 (0.72-3.13)
LRP	0.83 (0.36-1.89)	0.86 (0.50-1.46)	1.18 (0.70-1.97)	1.14 (0.46-2.81)	0.60 (0.25-1.46)	3.43 (1.48-7.97)^c^	2.89 (1.68-5.00)^c^	0.92 (0.51-1.68)	0.69 (0.28-1.72)	2.56 (1.41-4.64)^c^

Abbreviations: ADP, Alzheimer disease pathology; *APOE*, apolipoprotein E; BPSD, behavioral and psychological symptom of dementia; FDR, false discovery rate; HR, hazard ratio; LRP, Lewy body–related pathology; REM, rapid eye movement.

^a^ Indicates *P* < .05 meeting false discovery rate (FDR) threshold for lower HR.

^b^ Indicates *P* < .05 not meeting FDR threshold for lower HR.

^c^ Indicates *P* < .05 meeting FDR threshold for higher HR.

^d^ Indicates *P* < .05 not meeting FDR threshold for higher HR.

### Factors Associated With NPI-Q Scores

Among all participants across the sample, being younger was associated with higher NPI-Q composite score on longitudinal follow up (β = −0.07 [95% CI, −0.08 to −0.05]; *P* < .001). Other significant factors included female sex (β = −0.64 [95% CI, −0.95 to −033]; *P* < .001), lower educational level (β = −0.07 [95% CI, −0.12 to −0.02]; *P* = .004), executive initial symptoms relative to amnestic symptoms (β = 1.71 [95% CI, 1.17 to 2.25]; *P* < .001), and LRP vs ADP neuropathological group (β = 0.50 [95% CI, 0.03 to 0.98]; *P* = .04). When stratified by neuropathological groups, significant decreases in NPI-Q scores were seen for increasing age in the ADP (β = −0.08 [95% CI, −0.10 to −0.06]; *P* < .001), LRP (β = −0.09 [95% CI, −0.07 to −0.04]; *P* < .001), and ADP-LRP (β = −0.05 [95% CI, −0.07 to −0.02]; *P* < .001) groups. However, follow-up duration in the ADP (β = 0.32 [95% CI, 0.27-0.37]; *P* < .001), LRP (β = 0.29 [95% CI, 0.18-0.40]; *P* < .001), and ADP-LRP (β = 0.32 [95% CI, 0.26-0.38]; *P* < .001) groups and executive relative to amnestic symptoms in the ADP (β = 2.06 [95% CI, 1.25-2.87]; *P* < .001), LRP (β = 1.45 [95% CI, 0.18-2.71]; *P* < .001), and ADP-LRP (β = 1.43 [95% CI, 0.51-2.36]; *P* < .001) groups were significantly associated with higher NPI-Q scores. Significant negative associations between female sex and NPI-Q were also seen in the LRP (β = −1.69 [95% CI, −2.74 to −0.63]; *P* = .005) and ADP-LRP (β = −0.68 [95% CI, −1.19 to −0.17]; *P* = .01) groups. Neuropathology subgroup-specific longitudinal models for NPI-Q for these details is provided in eTable 3 in the Supplement.

## Discussion

In this study, we examined NACC data derived from 2422 participants enrolled at AD research centers across the US to investigate the clinical features at initial visit that are associated with the nature and severity of behavioral changes in individuals with ADP, LRP, and ADP-LRP. The results of this study indicate 2 key findings. First, among participants across the sample, initial cognitive symptoms are a significant clinical factor associated with specific BPSDs. Those with initial language symptoms had a lower risk of depression, visual hallucinations, and REM sleep BPSDs but a higher risk of personality changes. Participants with initial executive symptoms had a higher risk of developing most BPSDs, including apathy, delusions, disinhibition, visual hallucinations, personality changes, and REM sleep behavior changes. Participants with visuospatial initial symptoms had a higher risk of developing visual hallucinations and REM sleep behavior changes.

Second, the spectrum of BPSDs varies with the underlying neuropathology, with the presence of Lewy body pathology in ADP-LRP associated with a higher risk of BPSDs (delusions, auditory hallucinations, visual hallucinations, and REM sleep behavior changes) than ADP alone. In contrast, participants with LRP had a higher risk of visual hallucinations and REM sleep behavior changes than participants with ADP-LRP. Those with visuospatial symptoms had the highest risk of development of visual hallucinations in LRP and ADP-LRP, similar to that reported in prior studies.^46,47^ In addition, participants with LRP and ADP-LRP had a higher risk for auditory hallucinations and REM sleep behavior changes than those with ADP. Disinhibition, irritability, and agitation BPSDs were a higher risk among participants with ADP than among those with LRP.

Being younger was associated with a higher risk of most BPSDs and had the strongest association with the severity of NPI-Q score on longitudinal follow-up. Low educational level and male sex were also noted as other significant factors associated with NPI-Q severity among the mixed ADP-LRP and ADP groups and have been reported previously in clinical cohorts with mild cognitive impairment and early AD.^11,48^

There is increasing awareness that heterogeneity in initial cognitive symptoms among neurodegenerative diseases might be explained by differential effects on functional brain networks.^49^ Our results indicate that initial cognitive symptoms are also associated with specific BPSDs, consistent with the possibility of overlap in topography of neuronal dysfunction noted in cognitive phenotypes and underlying specific BPSDs. This outcome was observed with a higher risk of apathy, delusions, disinhibition, irritability, and personality changes among participants with initial executive symptoms,^50,51,52,53,54^ the highest risk of visual hallucinations among participants with initial visuospatial symptoms,^46,55^ and low risk of many BPSDs with initial language symptoms in participants with ADP and LRP.

Because the right prefrontal cortex is postulated to play a significant part in testing for reality-based beliefs^56^ and inhibition of contextually inappropriate affective and motor responses,^57^ it likely contributes to a higher risk of BPSDs with initial executive symptoms. However, because language symptoms are often lateralized to the left hemisphere in ADP,^58^ this theory likely is associated with a lower risk for delusions and hallucinations. Orbitomedial prefrontal cortex dysfunction and connections to the basal ganglia are thought to underpin apathy,^59^ which was noted to have a higher risk with executive symptoms and a lower risk with language symptoms in ADP. Another factor to consider could be environmental factors that an individual experiences with effects on agitation, disinhibition, and irritability,^60,61,62,63^ although the significance of some of our results, such as delusions, may vary based on the cultural background of the cohort.^64,65,66^ A higher risk of delusions among the ADP-LRP group compared with the LRP group also suggests a potential AD neuropathology–specific vulnerability as well. Higher educational level being associated with lower risk of hallucinations and irritability likely indicates cognitive reserve as a potential modifier of BPSDs.^60^ A summary of previous longitudinal studies in clinical AD^67^ reported no association between neuropsychiatric symptoms and *APOE ε4* status, consistent with our overall results.

### Strengths and Limitations

Strengths of our study include collation of longitudinal data, confirmation of underlying pathology, and delineation of initial cognitive symptoms as a window into early heterogeneity among regional cortical changes. However, the use of initial cognitive symptoms and clinician-documented BPSDs in the analysis is limited by the clinical subjectivity of assessment and documentation of initial cognitive and BPSD symptoms. Because the study cohort was limited to participants within the US, the results could differ in other nonwestern cultures.^65^ Given the prevalence of mixed dementia with vascular copathology, we evaluated the Hachinski Ischemic Scale scores across all groups and found them to be less than 2, which is below the threshold of 4 or less for discrimination of multi-infarct dementia pathology from AD.^68^ This may indicate relatively little contribution from multiple infarcts to the initial cognitive symptoms, although a contribution from small-vessel ischemic disease cannot be ruled out. Given the strengths and biases of the NACC database (eMethods in the Supplement), it is likely that the current results are generalizable to other prospective research cohorts, including clinical trials.

## Conclusions

The findings of this cohort study suggest that differences in neuropathology and the nature of the clinical phenotype, in addition to age, sex, and educational level, are important when exploring the impact of specific neural substrates of BPSDs in neurodegenerative diseases and evaluating clinical outcomes. Awareness of these associations could be helpful in dementia management.

## Notes

Supplement. **eMethods.** Details on Materials and Assessments **eTable 1.** Hachinski Ischemic Scale Clinical Score and Corresponding Vascular Pathology Frequency as Denoted in NACC at Autopsy **eTable 2.** Adjusted Hazards Ratio (HR) and 95% Confidence Intervals for Anxiety Across the Neuropathology Groups **eTable 3.** Pathology-Specific Longitudinal Models for NPI-Q **eFigure.** Flow Chart of Participant Selection
eReferences
