# Supplementary material for: Association of Variation in Behavioral Symptoms With Initial Cognitive Phenotype in Adults With Dementia Confirmed by Neuropathology
Source: JAMA Netw Open. 2022 Mar 3;5(3):e220729. doi: 10.1001/jamanetworkopen.2022.0729 (PMC8895258; doi:10.1001/jamanetworkopen.2022.0729)
Supplement: Supplement. — eMethods. Details on Materials and Assessments eTable 1. Hachinski Ischemic Scale Clinical Score and Corresponding Vascular Pathology Frequency as Denoted in NACC at Autopsy eTable 2. Adjusted Hazards Ratio (HR) and 95% Confidence Intervals for Anxiety Across the Neuropathology Groups eTable 3. Pathology-Specific Longitudinal Models for NPI-Q eFigure. Flow Chart of Participant Selection eReferences [file jamanetwopen-e220729-s001.pdf]

Supplementary Online Content

Pillai JA, Bena J, Rothenberg K, Boron B, Leverenz JB. Association of variation in behavioral symptoms with initial cognitive phenotype in adults with dementia confirmed by neuropathology. *JAMA Netw Open*. 2022;5(3):e220729. doi:10.1001/jamanetworkopen.2022.0729

eMethods. Details on Materials and Assessments

eTable 1. Hachinski Ischemic Scale Clinical Score and Corresponding Vascular Pathology Frequency as Denoted in NACC at Autopsy

eTable 2. Adjusted Hazards Ratio (HR) and 95% Confidence Intervals for Anxiety Across the Neuropathology Groups

eTable 3. Pathology-Specific Longitudinal Models for NPI-Q

eFigure. Flow Chart of Participant Selection

eReferences

This supplementary material has been provided by the authors to give readers additional information about their work.

© 2022 Pillai JA et al. *JAMA Network Open*.

eMethods. Details on Materials and Assessments

1. Details on NACC database.

2. Details on the predominant domain of cognitive symptom assessment

3. Correspondence between NPI-Q and UDS clinician determined behavioral symptoms

4. Details on neurocognitive tests used in the cohort

5. Summary of vascular co-pathology in the three neuropathology groups: ADP, LRP, and ADP-LRP.

1. Details on NACC database.

In brief, NACC data are collected by trained clinicians and clinic personnel from participants and their co-participants (usually a close friend or family member). The Uniform Data Set (UDS) is collected using a standardized evaluation of participants. All of the Alzheimer's disease center (ADC) personnel use the same standard forms and coding guidebooks that provide guidance on filling out the forms. The forms are developed by representatives from the ADCs themselves, so they are involved in the process of creating the standard forms. The UDS is longitudinal, and its protocol requires approximately annual follow-up for as long as the participant is able to be involved. Late-stage participants forced to drop out due to health may continue to be followed strictly for autopsy purposes. Determinations of cognitive status in NACC are based on a clinical consensus after review of all available information at each center.

As in many longitudinal neurodegenerative cohorts, there is a potential bias in participant recruitment and in their agreeing for final autopsy confirmation of diagnosis which are well recognized.

In addition other biases to consider include the following:

1. Selection bias of subjects who are best candidates for longitudinal follow up against subjects with rapidly progressive disease
2. A large proportion of patients with AD pathology in the elderly have mixed AD and vascular disease, the current neuropathology cohort we have assembled evaluated subjects without significant vascular pathology in order to evaluate the impact of AD pathology alone as much as possible on BPSD on initial cognitive phenotypes. Additional studies evaluating the role for mixed pathology of AD and vascular disease are needed in the future to complement the current analyses.
3. Co-occurring pathology including TDP-43 were not considered given the variability in its assessment in the early decades of the NACC cohort
4. Given potential differences among neuropathology analysis across centers, and evolution in diagnostic criteria there is also the concern for lack of uniform assessment of the vascular burden and LRP.

© 2022 Pillai JA et al. *JAMA Network Open*.

Given these caveats, the results are most generalizable to well characterized clinical cohorts of AD and DLB as one might find in clinical trial or in longitudinal follow up studies.

2. Details on the predominant domain of cognitive symptom assessment
NACCCOGF' is a NACC-derived variable documented by the clinician that was first recognized as a decline in the subject's cognition**.** There is no structured interview formalized for determining the answer to this specific variable but all clinicians use the same standard forms and coding guidebooks when filling out the data. According to the coding guidelines specified, the clinician's conclusions for the 'NACCCOGF' variable are expected to be based on information obtained through subject, co-participant, medical records, and/or observation. Additionally, other results from the neuropsychological test battery obtained (except for the Montreal Cognitive Assessment or Mini Mental Status Examination) and neuroimaging are not used to determine the answer for this question by the clinician.

3. Correspondence between NPI-Q and UDS clinician determine behavioral symptoms
NPI-Q and its list of symptoms have mostly corresponding NACC clinician determined variables with a few differences.

	UDS clinician determined	NPI-Q caregiver determined
Agitation	X	X
Anxiety	X	X
Apathy	X	X
Depression	X	X
Delusions	X	X
Disinhibition,	X	X
Auditory hallucinations	X	X (as hallucination)
Visual hallucinations	X	X (as hallucination)
Irritability	X	X
Personality change	X	

© 2022 Pillai JA et al. *JAMA Network Open*.

REM sleep behavior changes	X	
Euphoria/elation		X
Appetite/Eating		X
Night time behavior		X

‘Personality change’ is not listed in NPI-Q but has related variables “Euphoria/Elation, and Appetite/Eating”, which could be among the clinically significant change, is in the NACC clinician determined variables as ‘Personality Change’.

4. Details on neurocognitive tests used in the cohort

Attention was assessed using the Digit Span subtest (Digits Forward) from the Wechsler Adult Intelligence Scale (WAIS) *(1)* and the Trail-Making Test (TMT) Part A *(2)*. Executive functioning was quantified using WAIS Digit Span (Digits Backwards) *(1)*, Trail Making Test Part B, and the Digit Symbol-Coding subtest from the WAIS *(1, 2)*. Digits Backward Length (i.e., number of digits correctly repeated in reverse order) was also included as a variable of interest. Language related tests included in NACC were object naming assessed using the 30-item version of the Boston Naming Test (BNT) *(3)* and semantic fluency (animal/vegetable names generated in 60 seconds) *(4)*. The evaluation of memory included measures of verbal episodic memory (Wechsler Memory Scale, Logical Memory subtest) *(1)*. All four cognitive domains documented in the UDS were evaluated at the participant initial visit: attention, executive functioning, language, and memory. The number of subjects with specific tests of visuospatial function in the NACC data (Benton Figure copy and draw) were not adequate for detailed analysis as it was only provided from 2015 (version 3 of UDS).

5. Summary of vascular co-pathology in the three neuropathology groups: ADP, LRP, and ADP-LRP.

When comparing the Hachinski ischemic scale scores at clinical visit to determine the relative differences between the initial symptom groups on coexisting vascular symptoms, the mean scores across all groups were <2 and the scores were again not significantly different between the initial symptom groups. The Hachinski score at initial visit rather than the neuropathology of vascular disease burden at autopsy

© 2022 Pillai JA et al. *JAMA Network Open*.

was considered in this study, as the vascular burden could potentially change from initial visit to autopsy (e.g., stroke) for some subjects limiting their utility in evaluating their effect on symptoms at initial visit.

The NACC Neuropathology Data Set has undergone several revisions since its inception in 2002, the most substantial of which occurred in 2014. Over this time the data regarding characterization of the degree of vascular pathology has changed. In order to standardize the characterization of vascular pathology, in the current version, presence of one or more ischemic, hemorrhagic, or vascular pathology (including mild severity indicated for pathologies such as atherosclerosis) is documented as present, absent or unknown. Therefore this makes it difficult to accurately find correlation values between ante-mortem Hachinski score and categorical variable of presence versus absence of vascular pathology at autopsy. Even though the severity of vascular pathology is not able to be described based on current data, vascular pathology including mild severity indicated for pathologies such as atherosclerosis is present across all three pathologies but no correlations between them can be derived.

© 2022 Pillai JA et al. *JAMA Network Open*.

eTable 1. Hachinski Ischemic Scale Clinical Score and Corresponding Vascular Pathology Frequency as Denoted in NACC at Autopsy

	ADP (N=1,187)	LRP (N=331)	Mixed (N=904)			ADP (N=1,187)	LRP (N=331)	Mixed (N=904)
	**N**	**Statistics**	**N**	**Statistics**	**N**	**N**	**Statistics**	**N**
Hachinski score	1,162	0.90 ± 1.3	323	1.4 ± 1.8	870	0.82 ± 1.3	19.0	***<0.001***[a]
Vascular Disease	1,178	1,166 (99.0)	321	309 (96.3)	903	893 (98.9)	14.4	***<0.001***[c]

eTable 2. Adjusted Hazards Ratio (HR) and 95% Confidence Intervals for Anxiety Across the Neuropathology Groups

**Parameter**	**Level**	**HR (95% CI)**	**p-value**
Age at first visit		0.99 (0.97,1.00)	***0.030***
Sex, Female	Female	0.94 (0.72,1.22)	0.62
Education		1.03 (0.98,1.07)	0.24
APOE4	Yes	1.14 (0.85,1.51)	0.38
Initial Symptom	Executive vs. Amnestic	2.23 (1.49,3.33)	< ***0.001***
	Language vs. Amnestic	2.40 (1.67,3.46)	< ***0.001***
	Visuospatial vs. Amnestic	1.36 (0.66,2.81)	0.41
Group	LRP vs. ADP	0.79 (0.49,1.26)	0.32
	Mixed vs. ADP	1.23 (0.94,1.61)	0.12
	Mixed vs. LDP	1.56 (0.97,2.52)	0.067

© 2022 Pillai JA et al. *JAMA Network Open*.

eTable 3. Pathology-Specific Longitudinal Models for NPI-Q

		ADP		LRP		Mixed	
**Effect**	**Level**	**Estimate (95% CI)**	**p-value**	**Estimate (95% CI)**	**p-value**	**Estimate (95% CI)**	**p-value**
Age at First Visit, years		-0.08 (-0.10,-0.06)	***< 0.001***	-0.09 (-0.14,-0.04)	***< 0.001***	-0.05 (-0.07,-0.02)	***< 0.001***
SEX	Female	-0.36 (-0.79,0.07)	0.097	-1.69 (-2.74,-0.63)	***0.005***	-0.68 (-1.19,-0.17)	***0.013***
Education, years		-0.07 (-0.14,-0.00)	0.12	-0.13 (-0.27,0.02)	0.12	-0.05 (-0.13,0.03)	0.18
APOE4	Yes	0.06 (-0.38,0.50)	0.78	1.17 (0.08,2.26)	0.11	0.38 (-0.13,0.89)	0.21
Initial Symptom	Executive vs. Amnestic	2.06 (1.25,2.87)	***< 0.001***	1.45 (0.18,2.71)	***0.025***	1.43 (0.51,2.36)	***0.004***
	Language vs. Amnestic	-0.62 (-1.33,0.08)	0.25	-0.11 (-1.71,1.48)	0.89	-0.12 (-1.02,0.77)	0.89
	Visuospatial vs. Amnestic	0.25 (-1.08,1.58)	0.71	1.06 (-0.81,2.92)	0.60	0.57 (-0.75,1.88)	0.60
Time, years		0.32 (0.27,0.37)	***< 0.001***	0.29 (0.18,0.40)	***< 0.001***	0.32 (0.26,0.38)	***< 0.001***

© 2022 Pillai JA et al. *JAMA Network Open*.

eFigure. Flow Chart of Participant Selection

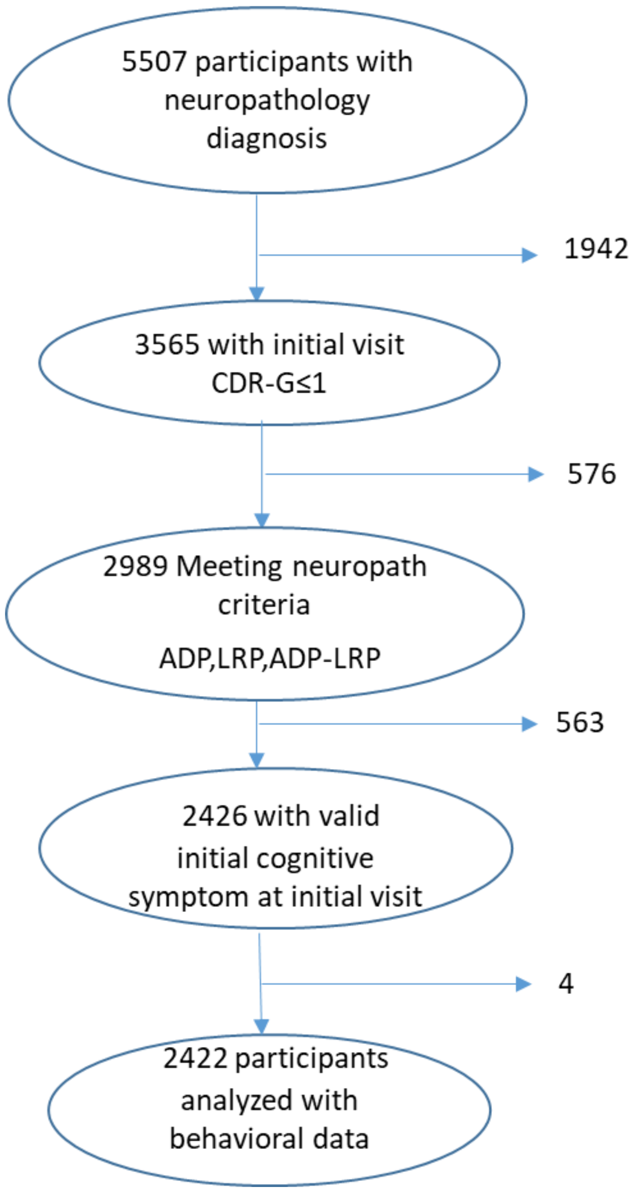

© 2022 Pillai JA et al. *JAMA Network Open*.

eReferences

1. Wechsler D. WAIS-R manual : Wechsler adult intelligence scale - revised. San Antonio: Psychological Corp. : Harcourt Brace jovanovich; 1981.
2. American Psychological A. Army Individual Test Battery2014.
3. Kaplan E, Goodglass H, Kaplan E, Segal O, Weintraub S.
4. Sherman EMS, Tan JE, Hrabok M. A compendium of neuropsychological tests administration, tests, norms, and commentary2020.

© 2022 Pillai JA et al. *JAMA Network Open*.
